# Inhibition of VE-PTP rejuvenates Schlemm’s canal in aged mice and acts via Tie2

**DOI:** 10.1371/journal.pone.0323615

**Published:** 2025-05-16

**Authors:** Sarthak Mishra, Ute Ipe, Astrid F. Nottebaum, Kevin G. Peters, Dietmar Vestweber

**Affiliations:** 1 Department of Vascular Cell Biology, Max Planck Institute for Molecular Biomedicine, Münster, Germany; 2 Aerpio Therapeutics, Inc., Blue Ash, Ohio, United States of America; Oregon Health and Science University, UNITED STATES OF AMERICA

## Abstract

**Purpose:**

Glaucoma is the leading cause of irreversible blindness worldwide and is associated with high intraocular pressure (IOP). Schlemm’s canal (SC), a hybrid vessel present in the anterior part of the eye, is known to control IOP by draining aqueous humor into the systemic circulation. Formation and function of SC is supported by the tyrosine kinase receptor Tie2. Likewise, inhibition of the vascular endothelial protein tyrosine phosphatase (VE-PTP), which associates with Tie2 has similar effects. However, VE-PTP also targets VE-cadherin and several other substrates. Here, we analyzed whether Tie2 is indeed the major substrate which is responsible for the role of VE-PTP in SC function. In addition, we analyzed the function of VE-PTP in SC of the aged eye in mice.

**Methodology:**

We tested the effects of the VE-PTP inhibitor AKB9778 and of VE-PTP gene inactivation on SC area and IOP in WT and in Tie2^iLEC/SC-KO^ and VE-cadherin-Y685F mutant mice.

**Results:**

Pharmacologic inhibition of VE-PTP with AKB9778 increased SC area only in mice expressing Tie2. The VE-cadherin-Y685F mutation had neither an effect on SC area nor on the effects of AKB9778 on SC formation. Induced VE-PTP gene inactivation in adult mice had similar effects as AKB9778. Furthermore, we could show that AKB9778 improved SC function in aged mice as judged by increasing SC area and lowering of IOP.

**Conclusion:**

Interference with VE-PTP function improves SC function in a strictly Tie2 dependent way and pharmacologic inhibition of VE-PTP with AKB9778 is a promising approach for improving SC function in the aged eye.

## Introduction

Glaucoma is one of the most prevalent causes of vision loss and blindness, affecting millions worldwide [1–3]. It is often associated with elevated intraocular pressure (IOP) due to obstruction of the aqueous outflow. The conventional outflow is mediated by the Schlemm’s canal and the trabecular meshwork which help in draining the aqueous humour produced by the ciliary body into the systemic circulation [4]. IOP elevation occurs when aqueous humour outflow is impaired [5]. Clinically, some drugs are on the market that reduce the IOP either by inhibiting the aqueous humour production rate or by increasing the outflow facility [4,6]. The latter can be achieved by targeting the unconventional outflow (uveoscleral pathway) or by supporting the function of the trabecular meshwork [4,6]. However, currently used medication rarely achieves a target IOP to eliminate disease progression.

Schlemm’s canal (SC) is a unique hybrid circular vascular structure harbouring both blood and lymphatic markers that runs circumferentially around the cornea. It originates from the blood vascular plexus and additionally acquires lymphatic characteristics during its development [7–9]. Its location in the anterior part of the eye helps to regulate IOP by draining the aqueous humour into the circulation. Dysfunction of SC leads to various ocular pathologies with an elevated IOP like glaucoma [10]. SC was found to be smaller in glaucomatous eyes [11,12]. Thus, proper functioning of SC is essential for maintaining the ocular homeostasis.

Angiopoetin-Tie2 signalling is important for the development and maintenance of Schlemm’s canal [10,13]. In mice, attenuation of Tie2 signalling was observed in aged animals and studies have shown association of aging with the progression of open angle glaucoma [10]. Partial or complete dysregulation of Tie2 leads to ocular pathologies associated with high IOP, like primary congenital glaucoma (PCG) which has been found to be associated with loss of function mutations in Tie2 or its primary ligand Angpt1 [13–15].

Activation of Tie2 may provide new therapeutic approaches to treat glaucoma by improving SC function. Past studies have shown the potential role of Tie2 activators like the antibody ABTAA [10] and a highly effective ANGPT1-mimetic fusion protein, Hepta-ANGPT1 [16], in improving SC function of mice. However, the invasive route of administering the drugs makes their use for therapeutical applications challenging.

Vascular endothelial tyrosine phosphatase (VE-PTP, also known as PTPRB), which is largely endothelial specific, influences endothelial junction stability by regulating Tie2 activity and VE-Cadherin adhesiveness [17–20]. Tie2 was the first identified substrate of VE-PTP [21]. Embryos homozygous for mutations of VE-PTP died at midgestation (by E10) due to severe vascular defects, demonstrating a critical role of VE-PTP in vessel maturation and remodeling [22,23]. Furthermore, we showed that VE-PTP controls blood vessel size and development by negatively regulating the tyrosine kinase Tie2 [24]. We also showed the effect of VE-PTP inhibition on increasing junctional stability via Tie2 [17].

Another important interacting partner of VE-PTP is the endothelial specific cell adhesion molecule, VE-cadherin [25], which provides stability to endothelial junctions. VE-PTP supports the function of VE-cadherin by regulating its phosphorylation state. Dissociation of VE-PTP from VE-cadherin is necessary for the opening of endothelial junctions during the induction of vascular permeability and leukocyte extravasation [18,26,27]. It dephosphorylates VE-cadherin at Tyr685 [18,26], thereby potentially counteracting endocytosis [28,29] and stabilizing junctions.

In cooperation with others, we have shown recently that VE-PTP is expressed in endothelial cells of the Schlemm’s canal [30]. In addition, we found that AKB9778, a specific inhibitor of VE-PTP, lowered the IOP in rabbit and mouse eyes and increased their outflow facility. Furthermore, SC area was increased in mice treated subcutaneously with AKB9778. Importantly, the IOP of ocular normotensive human patients suffering from diabetic retinopathy was found to be lowered when treated with AKB9778 subcutaneously [30]. Furthermore, topical AKB9778 (razuprotafib) as an adjunct to latanoprost therapy reduced IOP in patients with open angle glaucoma/ocular hypertension [31].These findings demonstrate the importance of VE-PTP for SC function in different species. However, whether VE-PTP acts strictly only via Tie2 was not shown and it remained to be shown whether inhibition of VE-PTP would be sufficient to reduce IOP and increase SC area in aged mice.

Here, we demonstrate that the VE-PTP inhibitor AKB9778 when administered as eyedrops increased SC area and reduced IOP in aged mice. The effects of AKB9778 on SC area in young mice were blocked by gene inactivation of Tie2, but were independent of VE-cadherin-Y685. Induced VE-PTP gene inactivation in SC endothelium had similar effects as AKB9778. We conclude that AKB9778 acts via VE-PTP/Tie2 and that topical ocular application of AKB9778 improves SC function and reduces IOP during aging.

## Results

### Inhibition of VE-PTP by AKB9778 in aged mice increases Schlemm’s canal area and reduces IOP

**Fig 1:**
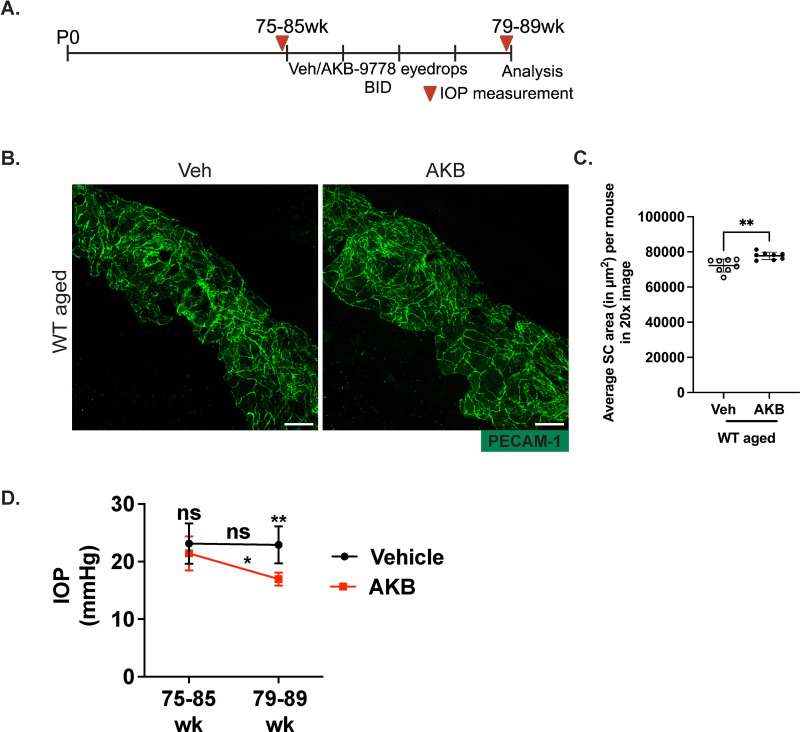
AKB9778 improves SC function in aged mice. A. Illustration of the topical ocular administration scheme of AKB9778 containing eyedrops or vehicle (twice daily) to 75–85-week-old mice and time points of IOP measurements. B. Confocal microscopy images of cornea whole mounts showing the SC area (green, PECAM-1) of vehicle or AKB9778 treated WT aged mice after 4 weeks of treatment (twice daily). Images shown are maximum intensity projections obtained from Z stacks with a step size of 0.89 µm, captured using a 20X objective. C. Quantification of SC area from vehicle or AKB9778-treated mice using Fiji software. Each dot represents the mean SC area of one mouse averaged over 12-16 20x images. D. IOP values measured before starting (75-85 wk) topical ocular eye drop treatment and at the end (79-89 wk) of the treatment using a rebound tonometer. n = 8 mice per group. Scale Bars: 50µm. p values for SC area were obtained using unpaired two tailed student’s T test and for IOP measurements using two-way ANOVA with Tukey’s multiple comparison; *P ≤ 0.05, **P ≤ 0.01.

In a previous study we showed that the VE-PTP inhibitor AKB9778 increases SC area in young, ocular normotensive WT mice. Based on this, we decided to test whether AKB9778 would improve SC function in aged mice. To this end, we treated eyes of 75–85-week-old WT mice (C57Bl/6) topically with AKB9778 containing eye drops or vehicle for 4 weeks twice daily (Fig 1A). IOP was measured before the first treatment and after the last treatment. Mice were then sacrificed and corneas were subjected to wholemount staining with antibodies against PECAM-1 as a marker for endothelial cell contacts. We detected a significant increase in SC area by 7.6% (±2.1%; P = 0.0025) in AKB9778 treated aged mice compared to vehicle treated control mice (Fig 1B and C). Moreover, we observed an increase in the number of Prox-1 positive SC endothelial cells, indicating rejuvenation of SC (S1 Fig A). In addition, we saw a significant reduction in apoptotic cells in the AKB treated group (S1 Fig B). Importantly, IOP of AKB9778 treated eyes was significantly reduced (Fig 1D). Thus, AKB9778 increases SC area and lowers IOP in aged mice.

### AKB9778 enlarges SC area independent of tyrosine 685 of VE-cadherin

**Fig 2:**
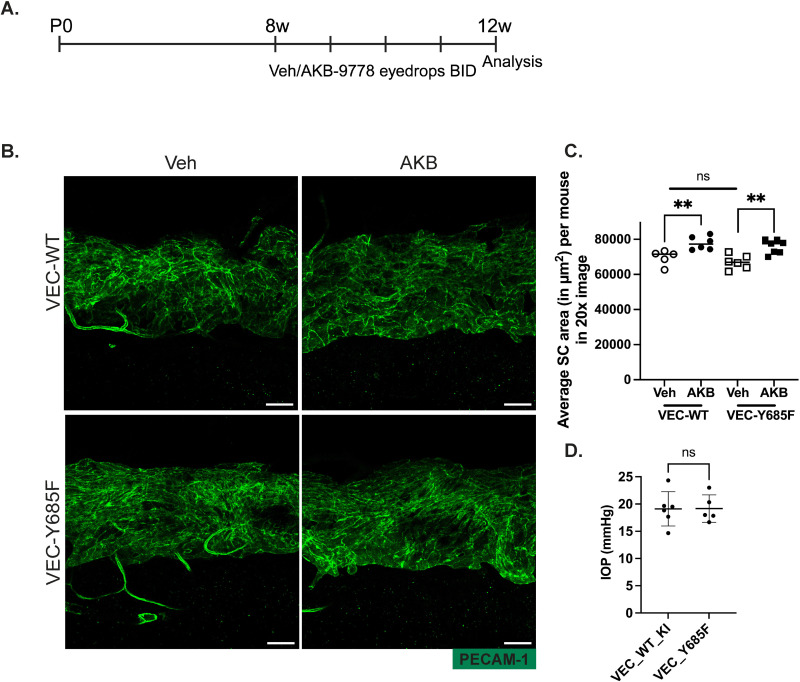
AKB9778 increases the SC area of VEC-Y685F mutant mice. A. Schematic representing timepoints of topical ocular application of AKB9778 containing eyedrops or vehicle. B. Confocal microscopy images of VEC-WT or VEC-Y685F-KI cornea whole mounts showing the SC area (green, PECAM-1) after 4 weeks treatment with Vehicle or AKB9778 eye drops (twice daily). Images shown are maximum intensity projections obtained from Z stacks with a step size of 0.90 µm, captured using a 20X objective. C. Quantification of SC areas shown in B using Fiji software. Each dot represents the mean SC area of one mouse averaged over 12-16 20X images. D. IOP values measured for VEC-WT-KI and VEC-Y685F mice using a rebound tonometer. n = 5 to 6 mice per group. Scale Bars: 50µm. p value for SC area was obtained using one-way ANOVA with Sidak’s multiple comparison, ns > 0.05, **P ≤ 0.01.

Next, we wanted to explore how AKB9778 mechanistically enlarges SC area and reduces IOP. We have shown previously that VE-PTP regulates VE-cadherin function and targets Y685 [18,25–27]. The role of this tyrosine for endothelial permeability [26] could potentially be relevant for fluid uptake into SC. Based on this, we tested whether Y685 of VE-cadherin contributes to SC area enlargement upon AKB9778 treatment. To assess this, we investigated knock in (KI) mice expressing either mutated VE-cadherin-Y685F (with tyrosine 685 being replaced by phenylalanine) or WT VE-cadherin and treated them twice daily for 4 weeks with AKB9778 containing eyedrops or vehicle (Fig 2A). At the end of the treatment, mice were sacrificed and corneas were subjected to wholemount staining with antibodies against PECAM-1 to visualize SC area. We observed a similar, significant increase in the SC area of AKB9778 treated VEC-Y685F and VEC-WT mice by 13% (±3.3%; P = 0.002) and 11.8% (±3.4%; P = 0.007), respectively, when compared to the vehicle treated mice (Fig 2B and C). We conclude, Y685 of VE-cadherin is not relevant for SC area enlargement stimulated by the VE-PTP inhibitor AKB9778. In line with these results, we found that IOP was similar in WT and VE-cadherin-Y685F mice (Fig. 2D), suggesting that phosphorylation of VE-cadherin at Y685 is not relevant for constitutive aqueous humor uptake in SC.

### AKB9778 induces SC area enlargement via Tie2

We then tested whether Tie-2 is the substrate of VE-PTP which is responsible for the increase in SC area induced by AKB9778. Although we know from previous work that VE-PTP inhibition leads to Tie-2 activation and that Tie-2 activation and VE-PTP inhibition each lead to SC area increase, this does not necessarily mean that Tie-2 is essential for the AKB9778 effect, especially in the light of many substrates that have been identified for VE-PTP [32].

Therefore, we induced Tie-2 gene inactivation of conditional knock out mice and tested whether this would block the effect of AKB9778 on SC. We first tested various tamoxifen-inducible Cre driver mouse lines for their efficiency to delete Tie-2 in SC. Surprisingly, two different Cre driver mouse lines, PDGFB-CreER^T2^ and Cdh5-CreER^T2^ when crossed with Tie-2^fl/fl^ mice and treated with tamoxifen daily for five days at the age of 7 weeks did not result in any significant reduction of Tie-2 expression in SC (not shown). Only when Prox1CreER^T2^ Χ Tie2^fl/fl^ mice (Tie2^iLEC/SC-KO^) were treated similarly with tamoxifen, Tie-2 expression was essentially absent in SC (S2 Fig B) whereas its expression in blood vessels above the SC was unaffected (S2 Fig A).

**Fig 3:**
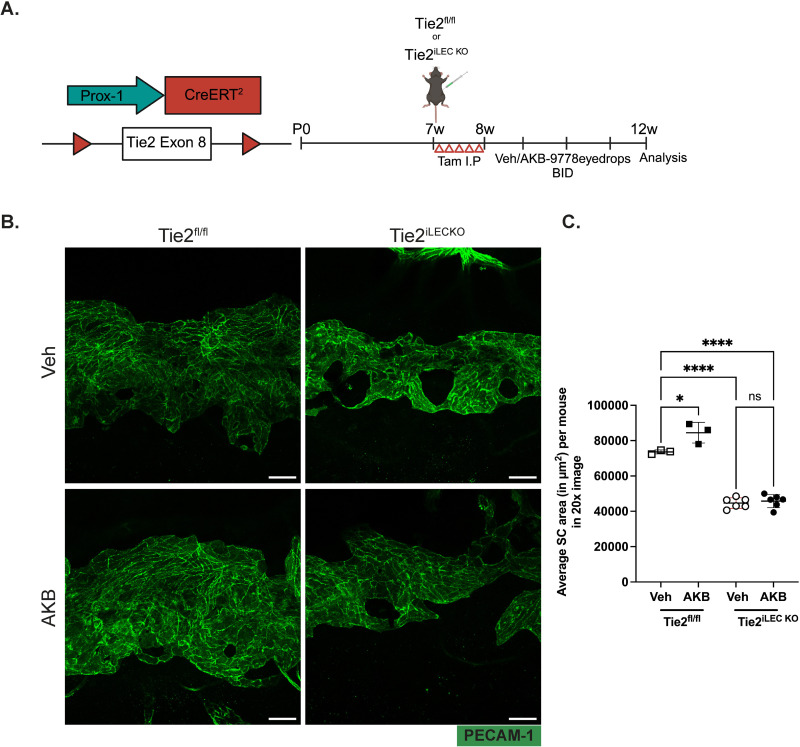
AKB9778 increases SC area via Tie2. A. Left: genetic diagram for Prox1 driven induced genetic inactivation of Tie2; Right: Scheme illustrating time points for Tamoxifen treatment for LEC and SC specific deletion of Tie2 and treatment with AKB9778 containing eyedrops or vehicle twice daily for 4 weeks. B. Confocal microscopy images of cornea whole mounts showing the SC area (green, PECAM-1) of Tie2^fl/fl^ and Tie2^iLEC/SC-KO^ mice after 4 weeks of topical ocular treatment with vehicle or AKB9778 (twice daily). Images shown are maximum intensity projections obtained from Z stacks with a step size of 0.90 µm, captured with a 20X objective. C. Quantification of SC areas shown in B using Fiji software. Each dot represents the mean SC area of one mouse averaged over 12-16 20X images. Scale Bars: 50µm. p values for SC area were obtained using one-way Anova with Tukey’s multiple comparison, ns > 0.05, *P ≤ 0.05, **** P ≤ 0.0001.

To determine the contribution of Tie2 to SC enlargement, Tie2^iLEC/SC-KO^ mice at the age of 7 weeks were treated with Tamoxifen on 5 consecutive days followed by ocular topical treatment with AKB9778 containing eye drops or vehicle twice daily for 4 weeks (Fig 3A). At the end of the treatment, mice were sacrificed, cornea whole mounts were stained for PECAM-1 and SC area was analyzed. Importantly, we found that AKB9778 only increased the area of SC in Tie-2^fl/fl^ mice, but showed no such effect in Tie2^iLEC/SC-KO^ mice (Fig 3B and C). Thus, Tie-2 is the essential substrate of VE-PTP, which is responsible for the effect of AKB9778 on SC area enlargement.

### SC specific VE-PTP KO increases SC area similar to AKB 9778 treatment

**Fig 4:**
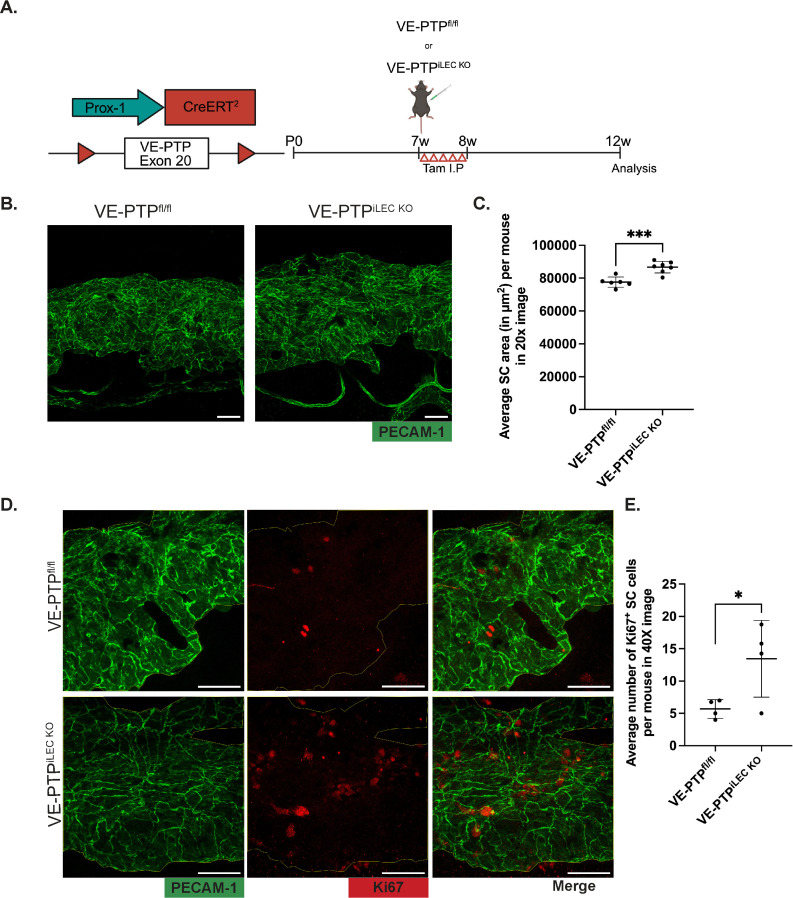
VE-PTP gene inactivation from SC leads to enlargement of SC area by increasing proliferation of SC endothelial cells. A. Left: genetic diagram for Prox1 driven induced genetic inactivation of VE-PTP; Right: Diagram depicting timepoints for Tamoxifen treatment for LEC and SC specific gene inactivation of VE-PTP from the SC and SC analysis. B. Confocal microscopy images of cornea whole mounts showing the SC area (green, PECAM-1) of VE-PTP^fl/fl^ and VE-PTP^iLEC/SC-KO^ mice after 4 weeks of VE-PTP depletion. Images shown are maximum intensity projections obtained from Z stacks with a step size of 0.89 µm, captured using a 20X objective. C. Quantification of SC areas shown in B) using Fiji software. Each dot represents the mean SC area of one mouse averaged over 12-16 20X images. D. Confocal microscopy images of cornea whole mounts of VE-PTP^fl/fl^ and VE-PTP^iLEC/SC-KO^ mice 4 weeks after tamoxifen treatment stained for PECAM-1 (green) and Ki67 (red). Images shown are maximum intensity projections obtained from Z stacks with a step size of 0.45 µm, captured using a 40X objective. E. Quantification of Ki67 + SECs. Each dot represents the mean number of Ki67 + SCECs of one mouse averaged over six 40X images. Scale Bars: 50µm. p values for SC area and Ki67 count were obtained using unpaired two tailed Student’s T test, *P ≤ 0.05, ***P ≤ 0.001.

Although AKB9778 is remarkably selective for VE-PTP, and the IC50 for many tyrosine phosphatases 100–10000 fold higher, there are some, which are efficiently inhibited with an only slightly higher IC50 [33,34]. One of them is DEP-1, which is also relevant for vessel development [35] and for vascular permeability [36]. Therefore, we decided to test whether the effect of AKB9778 on SC formation were indeed due to inhibiting VE-PTP. To this end, we interfered with VE-PTP in an alternative way and investigated VE-PTP conditional gene inactivated mice. By mating VE-PTP^fl/fl^ mice with Prox1-CreERT2 mice, we generated inducible VE-PTP^iLEC/SC-KO^ mice. These mice were treated at the age of 7 weeks with tamoxifen on 5 consecutive days and 4 weeks later they were sacrificed and cornea whole mount were stained for PECAM-1 and for Ki67 to analyze SC area and determine the number of proliferating endothelial cells. We found a significant increase in SC area of VE-PTP^iLEC/SC-KO^ mice when compared to VE-PTP^fl/fl^ mice (Fig 4B and C). Furthermore, we detected a higher number of Ki67 positive cells in SC of the conditional KO mice (Fig 4D and E). In agreement with these results, we found a robust increase in Tie2 phosphorylation in SC of VE-PTP^iLEC/SC-KO^ mice (S3 Fig A), and whole mount staining for VE-PTP documented the efficiency of VE-PTP deletion in SC (S3 Fig B). Collectively, these results show that it is interference with VE-PTP which leads to an increased area of SC. This effect seems at least in part be driven by proliferation of SC endothelial cells.

## Discussion

**Fig 5:**
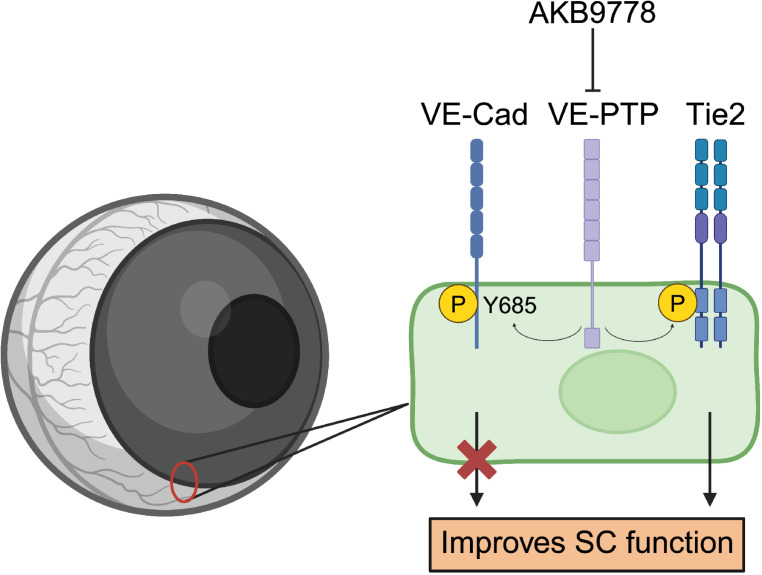
Mechanism by which the VE-PTP inhibitor AKB9778 improves SC function. AKB9778 improves SC function not by effects on VE-cadherin-Y685 phosphorylation, but by stimulating Tie2 activation which in turn increases cell proliferation and decreases apoptosis of endothelial cells of SC, thereby enlarging its filtration area.

This study explored the potential of an inhibitor of the phosphatase VE-PTP to have beneficial effects on SC and intraocular pressure (IOP) in aged mouse eyes. In addition, we investigated the molecular identity of direct and indirect targets of the inhibitor. We found that treatment of aged WT mice with AKB9778 eyedrops increased the area of SC and reduced IOP. Mechanistically, we showed that the beneficial effect of AKB9778 on SC was critically dependent on the expression of Tie-2 and was not related to Y685 on VE-cadherin, a second well studied *in vivo* substrate of VE-PTP in endothelium (Fig 5). Finally, induced gene inactivation of VE-PTP similarly increased SC area as the pharmacological inhibitor, confirming that it is VE-PTP which is responsible for the effects of the phosphatase inhibitor AKB9778 on SC *in vivo*. Increasing the area of SC was accompanied by increased proliferation of SC endothelial cells and reduced apoptosis.

Dysfunction in SC often occurs with aging and can contribute to the development of glaucoma [37–39]. Therefore, it is highly desirable to find ways to interfere with this process. In this context, it is remarkable that the VE-PTP inhibitor, administered as eyedrops, not only increased SC area but also reduced IOP in aged mice (75–85 weeks old). Although we have observed similar effects before in young mice (8 weeks of age) [30], it is not trivial that this could also be achieved in very old mice, since the expression levels of VE-PTP and Tie2 or the efficiency of downstream signaling could have deteriorated with age. Our results are in line with a recent study in human patients, where AKB9778 was able to reduce IOP when administered in adjunct to latanoprost [31]. In this context, it is interesting that stimulation of Tie2 in mice by intraocular injection of the monoclonal antibody ABTAA, which clusters the Tie2 ligand Angiopoietin-2, was able to increase the number of Ki67^+^ (proliferating) endothelial cells in SC of aged mice [10]. While this is in full agreement with our results, it is remarkable that no significant changes of SC area or IOP were observed in aged mice [10]. It was speculated that the lack of significant reduction of IOP might have been due to reduced aqueous humor production in aged mice or due to the fact that the antibody ABTAA was only administered for 2 weeks [10]. The latter argument could indeed be a potential explanation for the difference to our results since we applied AKB9778 for 4 weeks. In addition, it is presently not known how ABTAA and AKB9778 compare with respect to the strength of Tie-2 activation. Interestingly, we also observed that the number of caspase 3^+^ (apoptotic) endothelial cells in aged SC was reduced by treatment with AKB9778. As aged glaucoma or other types of glaucoma are often associated with high IOP [40–42], the effect of AKB9778 on IOP of aged mouse eyes and the recent phase 2 study in humans [31] make it a potentially promising candidate for further trials on non-invasive treatment of glaucoma.

Interestingly, it was reported that SC area is not different in old and young mice and this is in agreement with our results when we compare vehicle treated young and old mice from different experiments. In addition, it was shown that – in contrast to humans – in mice, aqueous humor outflow does not decrease with age [43], which is in line with tracer distribution studies [44]. It was suggested that this is because uveoscleral aqueous humor outflow has a greater role in mice, thereby compensating for the dysfunctional drainage through SC, unlike in humans [45]. However, this was recently shown to be incorrect [46]. Mice and humans are in fact similar with respect to conventional versus unconvential outflow usage [47]. Considering all this, it is remarkable that AKB9778 could increase SC area and reduce IOP in mice, as we show here.

It is a possibilty, that AKB9778 could have effects on distal outflow, in addition to the effects we see here on SC. However, since we see similar effects by Prox1-CreER^T2^ driven VE-PTP gene inactivation, we can be sure that these are due to VE-PTP in SC endothelial cells. Whether this SC specific gene deletion then has additional indirect effects on TM and distal vessels is presently unknown. While it is clear that interference with VE-PTP improves SC structure as is based here on flat mounts, we did not direcly measure outflow facility or perform more detailed morphological studies of outflow structures other than SC flat mounts.

VE-cadherin Y685 is a substrate for VE-PTP and phosphorylation of this site contributes to permeability induction by reducing endothelial junctional integrity in inflamed blood vessels [26]. Thus, inhibiting VE-PTP function could perhaps support fluid uptake through endothelial cell contacts which could improve outflow efficiency of the eye. Therefore, Y685 of VE-cadherin would have been an attractive additional VE-PTP target to explain the beneficial effects of AKB9778 on SC. However, we found that mice expressing the VE-cadherin Y685F mutant instead of WT VE-cadherin had similar IOP levels, arguing that constitutive aqueous humor uptake into SC is independent of Y685 phophorylation of VE-cadherin. In line with this, we found that AKB9778 increased SC area as efficiently in WT mice as in VE-cadherin-Y685F mice. In contrast, the AKB9778 effects were completely blocked in mice gene inactivated for Tie2 in endothelial cells of SC. Thus, Tie2 is the substrate which is strictly required for the effects which we observed for the AKB9778 VE-PTP inhibitor on SC endothelium.

Although AKB-9778 is a highly specific inhibitor of VE-PTP with an IC50 which is magnitudes lower than for many other phosphatases [33,34], some closely related phosphatases, such as DEP-1 can be inhibited by AKB9778 almost as efficiently as VE-PTP [33,34,48,49]. Therefore, it was important to verify that the AKB9778 effects, we observed for SC, were indeed mediated by inhibiting VE-PTP and not other phosphatases. Importantly, we could demonstrate this by Prox1-Cre-ER^T2^-driven inactivation of the VE-PTP gene, which led to SC area enlargement similar to the results we obtained with AKB9778.

In contrast to the above findings, it was previously reported that gene inactivation of VE-PTP postnatally induced by three daily tamoxifen injections starting at P0 did not affect SC area at P14 [16]. The apparent discrepancy to our results may be explained by several arguments. First, different Cre drivers were used. The cited study used Cdh5 CreER^T2^ as a driver for genetic deletion, while we used Prox1-CreER^T2^. Second, the time course of the experiments was different, the mentioned study analyzed the anatomy of SC only 10 days after gene deletion, whereas we did the analysis 4 weeks after the last tamoxifen injection. Third, the study mentioned above analyzed postnatal stages whereas we analyzed young adult mice. Fourth, we documented the loss of VE-PTP protein in SC by antibody staining, while such an analysis was not reported in [16]. Interestingly, VE-PTP gene inactivation did show an effect on superficial limbal vasculature in the eye, suggesting effects based on interference with VE-PTP expression in other vessels than the SC [16]. While this is indeed an interesting finding, we can exclude that our effects can be explained this way, since we used a Prox1-CreER^T2^ driver which would induce gene inactivation only in lymphatics (where VE-PTP is not expressed) and in SC endothelium. Our results are also in agreement with another study, which reported defects in SC development in mice carrying only one allele of Tie2 which was rescued by haploinsufficiency of VE-PTP [50].

Together, our findings demonstrate that VE-PTP is targeted in SC endothelium by the inhibitor AKB9778. This interaction causes SC enlargement and reduction of IOP in aged mouse eyes. These effects require the expression of and are mediated by Tie-2. We conclude that AKB9778 improves SC function via targeting VE-PTP/Tie2 and support the notion that this agent is a promising drug for treating age related open-angle glaucoma.

## Methods

### Mice

All mice used in this study were on the C57BL/6Jrj genetic background. Wild-type C57BL/6Jrj mice of both genders aged between 75–85 weeks were used for the study.

VE-PTP^fl/fl^ [17] mice were bred with Prox1-CreER^T2^ mice [51] (a kind gift from Taija Mäkinen) to create Tamoxifen-inducible lymphatic endothelial cell (LEC)-specific and SC-specific VE-PTP knockout mice (VE-PTP^iLEC/SC-KO^). For VE-PTP deletion, 7-week-old mice were injected intraperitoneally with 3 mg tamoxifen (T5648, Sigma‐Aldrich) dissolved in 3% ethanol and 97% peanut oil daily for five consecutive days. Analysis was done 4 weeks later.

By mating Tie2^fl/fl^ mice with Prox1-CreER^T2^ mice Tamoxifen-inducible lymphatic endothelial cell-specific and SC specific Tie2 knockout mice (Tie2^iLEC/SC-KO^) were generated. For Tie2 deletion, 7-week-old mice were injected intraperitoneally with 2 mg tamoxifen (T5648, Sigma‐Aldrich) dissolved in 2% ethanol and 98% peanut oil daily for five consecutive days. Analysis was done 4 weeks later.

VE-cadherin tyrosine 685 mutant knock-in mice were generated as described previously [26].

### AKB9778 eyedrops treatment

AKB9778 containing eye drops (40mg/ml) or vehicle were received from Aerpio pharmaceuticals (Cincinnati, OH, USA). For treatment, mice were briefly anesthetized using an Isoflurane evaporator and 5 µl (adult mice) or 10 µl (aged mice) of eyedrop solution were applied twice daily for 4 weeks to each eye.

### IOP measurements

Aged wild-type C57BL/6 mice treated with AKB9778-containing eyedrops or vehicle were subjected to IOP measurement at the start and at the end of the treatment. Measurements were done using a rebound tonometer (iCare TonoLab). Briefly, mice were anesthetized with ketamine (60 mg/kg) and xylazine (6 mg/kg). IOP was measured as soon as the mice stopped moving (light sleep).

The tonometer gives a value which is an average of 6 readings. For each eye, 3 values were taken meaning 18 readings per eye. 6 values from both eyes of the mice were then averaged and considered as the final IOP.

### Whole mount staining of the eye

Whole mount staining for Schlemm’s canal detection was done as previously described [30]. Mice were euthanized using CO_2_, followed by perfusion with 1% PFA via the left ventricle. After enucleation eyeballs were fixed in 4% PFA for 30 minutes at RT, followed by incubation in fixation buffer (1% PFA, 0.1% Triton X-100, and 0.1% NP-40 in PBS) for 30 minutes at RT. After 3 washes in PBS for 5 minutes, corneas were isolated, permeabilized and blocked in blocking buffer (5% donkey serum, 0.2% BSA, and 0.2% Triton X-100 in PBS) for 3 hours at RT. Subsequently, the samples were incubated with primary antibodies in blocking buffer overnight at RT. Then, samples were washed in washing buffer (0.3% Triton X-100 in PBS) 6 times for 15 minutes at RT and were incubated with secondary antibodies in blocking buffer overnight at RT, followed by 6 washes in washing buffer for 30 minutes at RT before mounting in DAKO fluorescent mounting medium. Analysis was done using a confocal laser scanning microscope (Zeiss LSM 880) with online fingerprinting mode (ZEN software). Processing was done in ZEN software while analysis was done using Fiji. For detection of phosphotyrosine, 1 mM sodium orthovanadate was added to all buffers.

The following primary antibodies were used for immunofluorescence staining at the indicated concentrations: anti-VE-PTP (rat-anti-mouse-VE-PTP mAb 109.1, 10 µg/mL) [22], anti-VE-cadherin (goat polyclonal, R&D #AF1002, 5 µg/mL), anti-Prox1 (rabbit polyclonal, ReliaTech #102-PA32AG, 2.5 µg/mL), anti-Prox1 (goat-anti-human-Prox1, R&D, AF-2727, 2 µg/mL); anti-PECAM-1 (rat-anti-mouse PECAM-1 clone 1G5.1 (3 µg/mL) + clone 5D2.6 (1 µg/mL) [52], anti-Ki67 (rabbit monoclonal, clone SP6, Abcam #ab16667, 0.3 µg/mL), anti-Tie2 (goat polyclonal, R&D #AF762, 5 µg/mL); and anti-phospho-Tie2 (rabbit polyclonal, R&D #AF2720, 10 µg/mL). The following Alexa Fluor labelled secondary antibodies (all from Thermo Fisher Scientific) were used at a concentration of 2 µg/mL: donkey-anti-rat-IgG-Alexa-488 (A-21208); donkey-anti-goat-IgG-Alexa-568 (A-11057); donkey-anti-goat-IgG-Alexa-647 (A-10042); and donkey-anti-rabbit-IgG-Alexa-568 (A-21447).

### Image analysis

Images from antibody-stained cornea whole mounts were captured using a Zeiss LSM 880 confocal microscope with various objectives (20X, 40X, 63X) and a pinhole of 1 airy unit. Using Zen software, maximum intensity projections of all images were prepared and analyzed in Fiji as previously described [30]. For the quantification of SC area, PECAM-1^+^ area was calculated from 20X images. To quantify the proliferative status of SC, Ki67^+^ cells within the PECAM-1^+^ area were counted.

### Study approval

Animal care and experimental procedures were performed according to the German Animal Protection Act (Deutsches Tierschutzgesetz) and were approved by the Landesamt für Natur, Umwelt und Verbraucherschutz Nordrhein-Westfalen, Germany, protocol number 81-02.04.2019.A443.

### Statistics

GraphPad Prism 9 software was used for the analysis of the datasets. Comparisons between 2 groups were performed using two tailed unpaired Student’s t‐test. Comparisons among three groups were performed using one‐way ANOVA followed by Tukey’s multiple comparison test or sidak’s multiple comparison test. For IOP values, two-way ANOVA with Tukey’s multiple comparison test was performed. P values are indicated by asterisks: *P < 0.05, **P < 0.01. Results are shown as mean ± SD.

## Supporting information

S1 Fig Number of Prox-1^+^ cells and activation of caspase-3 in aged SC upon AKB treatment. **A.** Confocal microscopy images of cornea whole mounts showing the SC (green, PECAM-1) and Prox-1 expression (in magenta) of Vehicle or AKB9778 treated WT aged mice after 4 weeks of treatment (twice daily). Graph beside the staining depicts the quantification of Prox-1 positive cells (Prox-1^+^) per 40X image using Fiji software. Each dot represents the average value taken from eight 40X images per mouse. B. Confocal microscopy images of cornea whole mounts showing the SC (green, PECAM-1) and expression of active caspase-3 (in red) of Vehicle or AKB9778 treated WT aged mice after 4 weeks of treatment (twice daily). Graph beside the staining depicts the quantification of active caspase-3 expressing cells (caspase-3^+^) per 40X image using Fiji software. Each dot represents the average value taken from eight 40X images per mouse. Scale Bars: 20µm. p values for Prox-1^+^ and active caspase-3^+^ count were obtained using unpaired two tailed Student’s T test, *P ≤ 0.05, **P ≤ 0.01.
(PDF)

S2 Fig SC specific deletion of Tie2 using Prox-1CreER^T2^ mice. **A.** Confocal microscopy images of cornea whole mounts showing the vessels located above SC (green, PECAM-1) and Tie2 expression (in red) of both Tie2^fl/fl^ and Tie2^iLEC/SC-KO^ mice after tamoxifen treatment (Scale Bars: 50µm). B. Confocal microscopy images of cornea whole mounts showing the SC (green, PECAM-1) and Tie2 expression (in red) of both Tie2^fl/fl^ and Tie2^iLEC/SC-KO^ mice after tamoxifen treatment (Scale Bars: 50µm).
(PDF)

S3 Fig SC specific deletion of VE-PTP using Prox-1CreERT2 mice. **A.** Confocal microscopy images of cornea whole mounts showing the SC (green, PECAM-1), Tie2 expression (in magenta) and Tie2 phosphorylation (in red) of both VE-PTP^fl/fl^ and VE-PTP^iLEC/SC-KO^ mice after tamoxifen treatment (Scale Bars: 50µm). B. Confocal microscopy images of cornea whole mounts showing the SC (magenta, VE-cadherin), VE-PTP expression (green) of VE-PTP^fl/fl^ and VE-PTP^iLEC/SC-KO^ mice after tamoxifen treatment (Scale Bars: 50µm).
(PDF)

S1 Data (XLSX)
