# Supplementary figures and images for: Inhibition of VE-PTP rejuvenates Schlemm’s canal in aged mice and acts via Tie2
Source: PLoS One. 2025 May 16;20(5):e0323615. doi: 10.1371/journal.pone.0323615 (PMC12084045; doi:10.1371/journal.pone.0323615)

Figure S1

A.

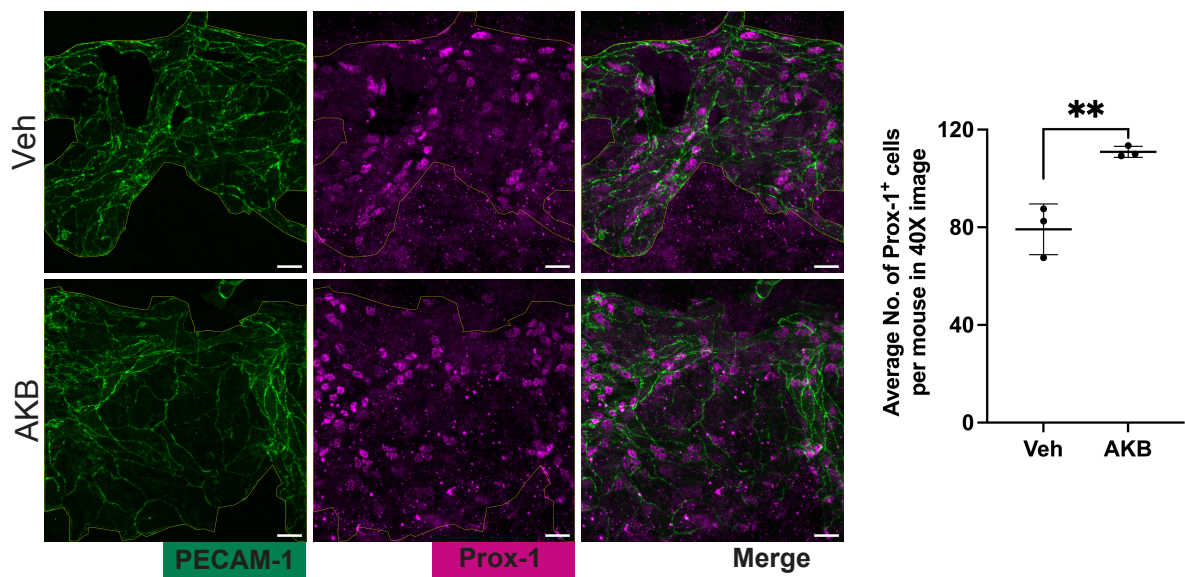

B.

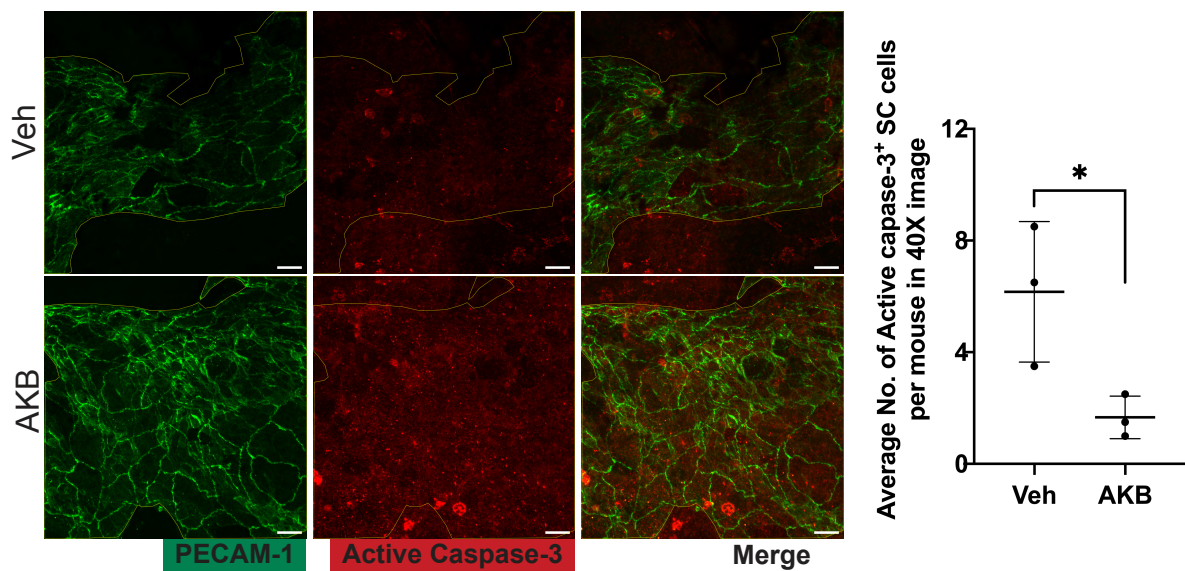

Supplement: S1 Fig — A. Confocal microscopy images of cornea whole mounts showing the SC (green, PECAM-1) and Prox-1 expression (in magenta) of Vehicle or AKB9778 treated WT aged mice after 4 weeks of treatment (twice daily). Graph beside the staining depicts the quantification of Prox-1 positive cells (Prox-1+) per 40X image using Fiji software. Each dot represents the average value taken from eight 40X images per mouse. B. Confocal microscopy images of cornea whole mounts showing the SC (green, PECAM-1) and expression of active caspase-3 (in red) of Vehicle or AKB9778 treated WT aged mice after 4 weeks of treatment (twice daily). Graph beside the staining depicts the quantification of active caspase-3 expressing cells (caspase-3+) per 40X image using Fiji software. Each dot represents the average value taken from eight 40X images per mouse. Scale Bars: 20µm. p values for Prox-1+ and active caspase-3+ count were obtained using unpaired two tailed Student’s T test, *P ≤ 0.05, **P ≤ 0.01. (PDF) [file pone.0323615.s001.pdf]

Figure S2

A.

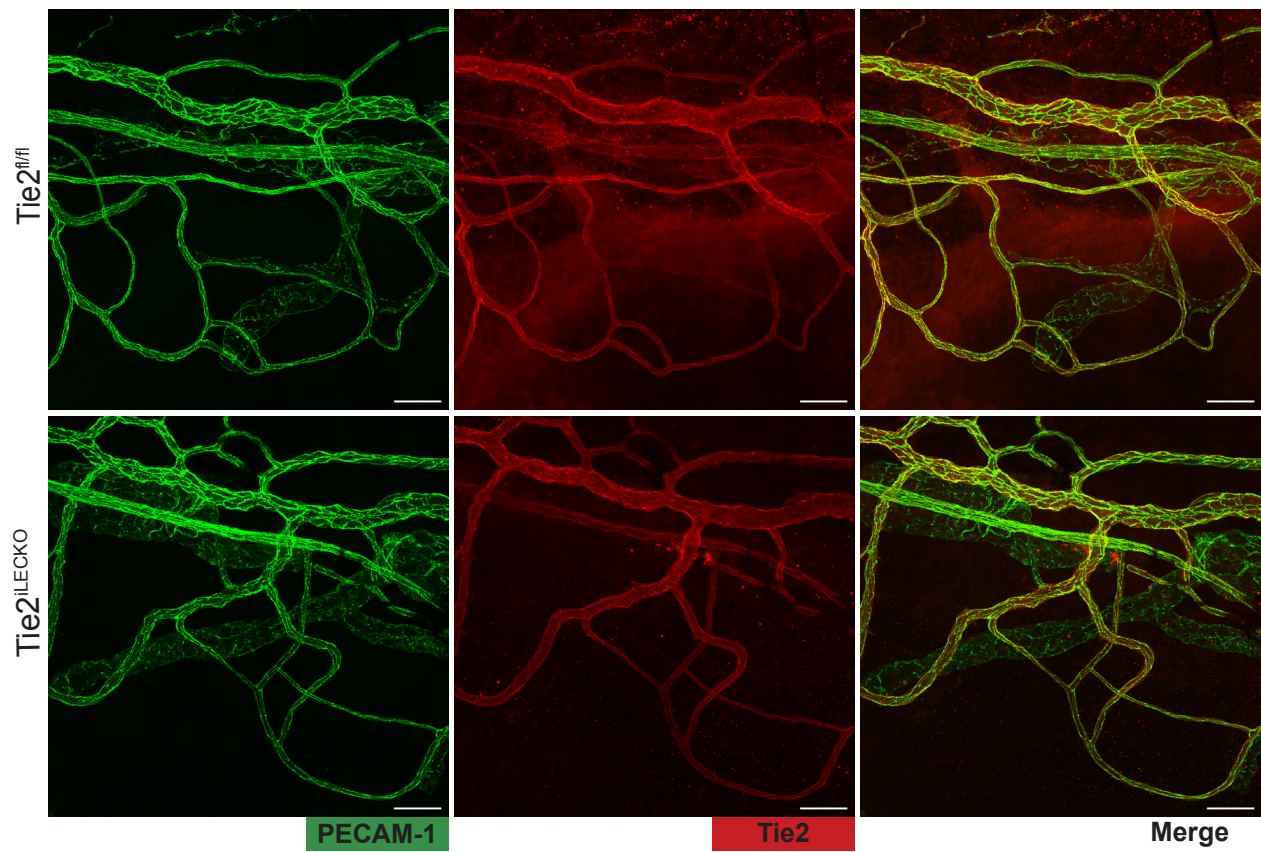

B.

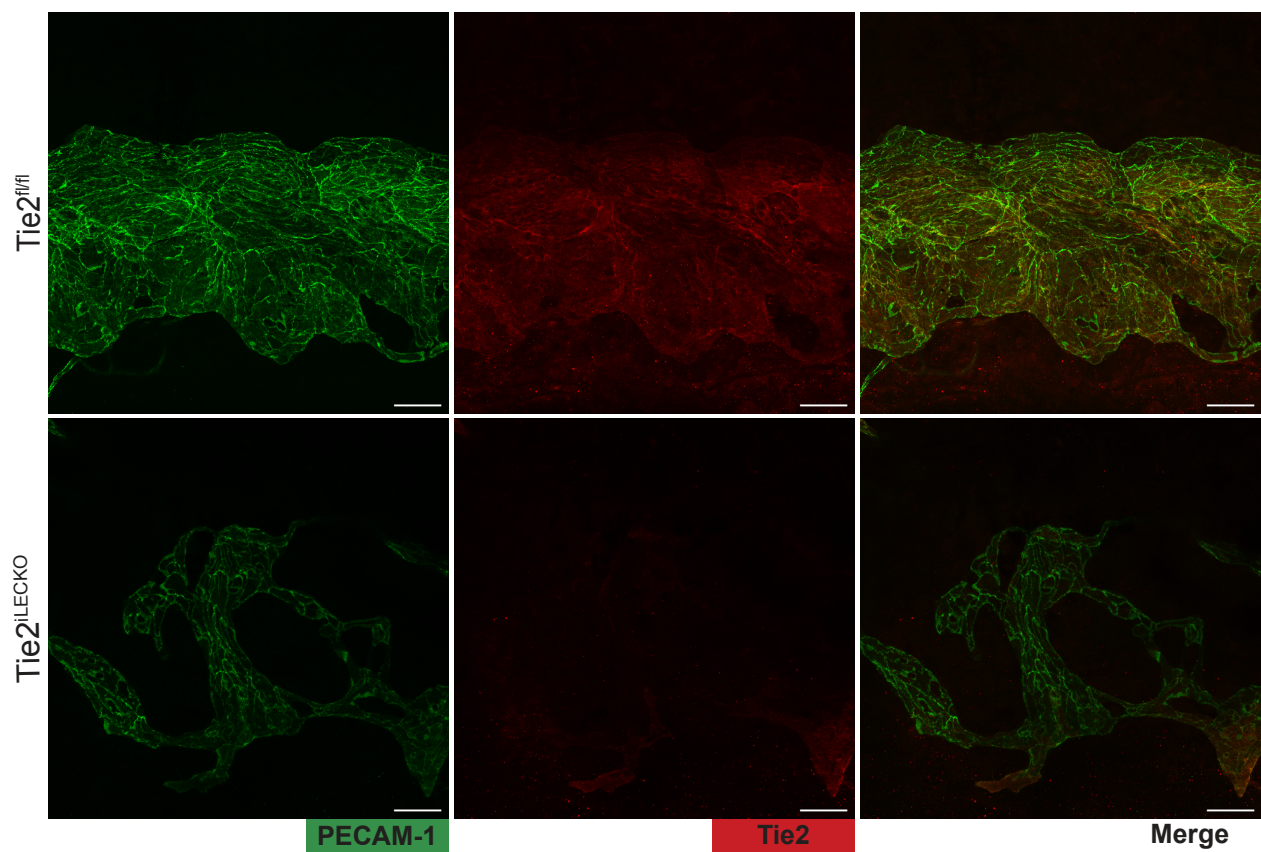

Supplement: S2 Fig — A. Confocal microscopy images of cornea whole mounts showing the vessels located above SC (green, PECAM-1) and Tie2 expression (in red) of both Tie2fl/fl and Tie2iLEC/SC-KO mice after tamoxifen treatment (Scale Bars: 50µm). B. Confocal microscopy images of cornea whole mounts showing the SC (green, PECAM-1) and Tie2 expression (in red) of both Tie2fl/fl and Tie2iLEC/SC-KO mice after tamoxifen treatment (Scale Bars: 50µm). (PDF) [file pone.0323615.s002.pdf]

Figure S3

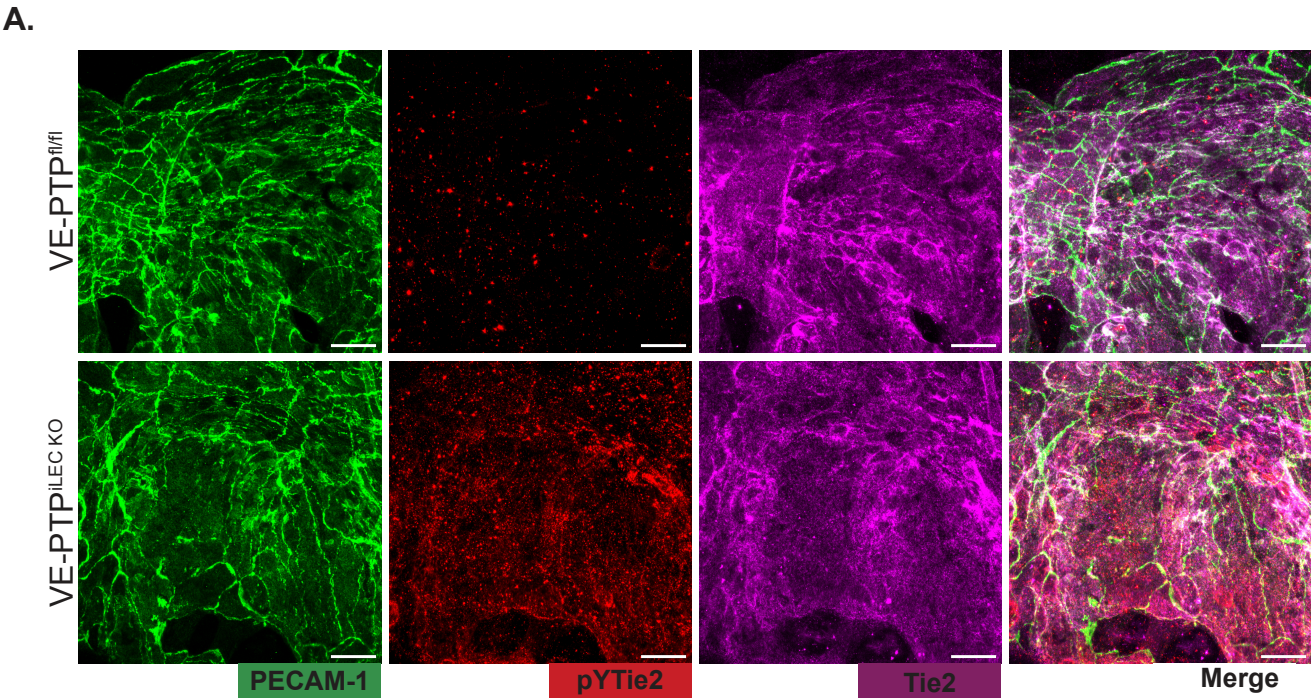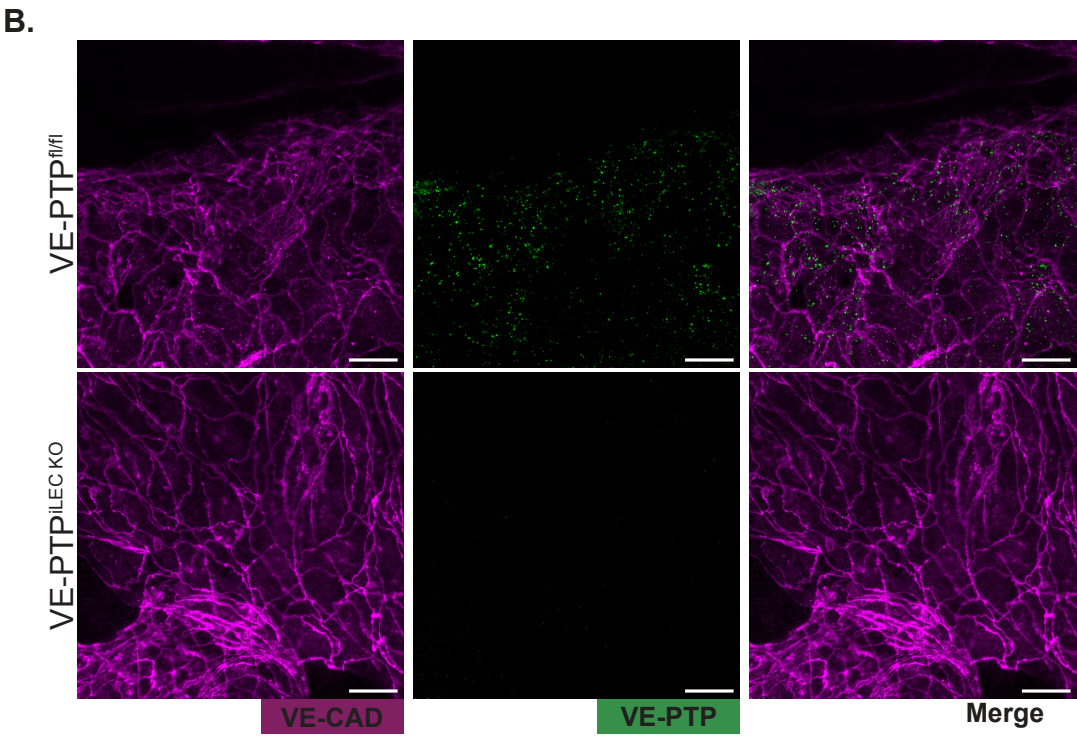

Supplement: S3 Fig — A. Confocal microscopy images of cornea whole mounts showing the SC (green, PECAM-1), Tie2 expression (in magenta) and Tie2 phosphorylation (in red) of both VE-PTPfl/fl and VE-PTPiLEC/SC-KO mice after tamoxifen treatment (Scale Bars: 50µm). B. Confocal microscopy images of cornea whole mounts showing the SC (magenta, VE-cadherin), VE-PTP expression (green) of VE-PTPfl/fl and VE-PTPiLEC/SC-KO mice after tamoxifen treatment (Scale Bars: 50µm). (PDF) [file pone.0323615.s003.pdf]
